# Syngeneic Mesenchymal Stem Cells Reduce Immune Rejection After Induced Pluripotent Stem Cell-Derived Allogeneic Cardiomyocyte Transplantation

**DOI:** 10.1038/s41598-020-58126-z

**Published:** 2020-03-12

**Authors:** Shohei Yoshida, Shigeru Miyagawa, Toshihiko Toyofuku, Satsuki Fukushima, Takuji Kawamura, Ai Kawamura, Noriyuki Kashiyama, Yuki Nakamura, Koichi Toda, Yoshiki Sawa

**Affiliations:** 1 Department of Cardiovascular Surgery, Osaka University Graduate School of Medicine, 2-2 Yamadaoka, Suita Osaka, 565-0871 Japan; 2 Department of Immunology and Molecular Medicine, Osaka University Graduate School of Medicine, Suita Osaka, Japan

**Keywords:** Cardiac regeneration, Stem-cell research

## Abstract

Avoiding immune rejection after allogeneic induced pluripotent stem cell-derived cardiomyocyte (iPSC-CM) transplantation is a concern. However, mesenchymal stem cells (MSCs) can suppress immune rejection. To determine whether MSC co-transplantation can reduce immune rejection after allogeneic iPSC-CM transplantation, the latter cell type, harbouring a luciferase transgene, was subcutaneously transplanted alone or together with syngeneic MSCs into BALB/c mice. Bioluminescence imaging revealed that MSC co-transplantation significantly improved graft survival (day 7: iPSC-CMs alone 34 ± 5%; iPSC-CMs with MSCs, 61 ± 7%; *P* = 0.008). MSC co-transplantation increased CD4 + CD25 + FOXP3 + regulatory T cell numbers, apoptotic CD8-positive T cells, and IL-10 and TGF-beta expression at the implantation site. Analysis using a regulatory T cell depletion model indicated that enhanced regulatory T cell populations in the iPSC-CM with MSC group partially contributed to the extended iPSC-CM survival. Further, MSCs affected activated lymphocytes directly through cell–cell contact, which reduced the CD8/CD4 ratio, the proportion of Th1-positive cells among CD4-positive cells, and the secretion of several inflammation-related cytokines. Syngeneic MSC co-transplantation might thus control allogeneic iPSC-CM rejection by mediating immune tolerance via regulatory T cells and cell–cell contact with activated lymphocytes; this approach has promise for cardiomyogenesis-based therapy using allogeneic iPSC-CMs for severe heart failure.

## Introduction

Heart failure is still associated with a high mortality rate worldwide, despite significant advances in medical treatment and technology. Therefore, it is critical to apply new concepts for the development of novel therapeutic alternatives^1,2^. In the last decade, stem cell therapy using bone marrow progenitor, cardiac, and somatic stem cells has been studied in clinical settings^3–6^. Unfortunately, however, these treatments have limited efficacy, likely because they depend primarily on paracrine effects induced by the transplanted cells, and thus the recovery of cardiomyocyte numbers is not sufficient. Recently, induced pluripotent stem cell (iPSC)-derived cardiomyocytes (iPSC-CMs) were proposed as a cell source for cardiomyogenesis-based therapy, which could be an alternative candidate treatment to several previous stem cell applications to achieve the benefit of increasing the number of functioning cardiomyocytes, as well as paracrine effects^7–9^. Although regenerative therapy using allogeneic iPSCs has more merit in terms of safety, cost, and preparation, compared to the use of autologous iPSCs, allogeneic cell transplantation is significantly hampered by immune rejection^10^. Several immunosuppressive agents that can result in renal failure, infectious disease, and malignancy are required for allogeneic cell transplantation, even though this approach might not have the potential to provide the same therapeutic efficacy as organ transplantation. Therefore, methods other than immunosuppressive agents should be studied to increase transplanted cell survival without the aforementioned side effects.

Bone marrow-derived mesenchymal stem cells (MSCs) were reported to have profound immunomodulatory properties by changing cell numbers and functions in the immune system^11–13^. In fact, MSC-based therapy was shown to have a significant therapeutic effect on a variety of human diseases including multiple sclerosis, inflammatory bowel disease, autoimmune encephalomyelitis, rheumatoid arthritis, systemic lupus erythematosus, and graft versus host disease^14–21^. However, the detailed mechanism underlying the immunosuppressive effect of MSCs has not been fully uncovered. In this study, we demonstrated that the co-transplantation of syngeneic MSCs enhances the survival of allogeneic transplanted iPSC-CMs through the induction of regulatory T cells (Tregs) and cell–cell contact in mice.

## Results

### Differentiation of cardiomyocytes derived from iPSCs

**Figure 1:**
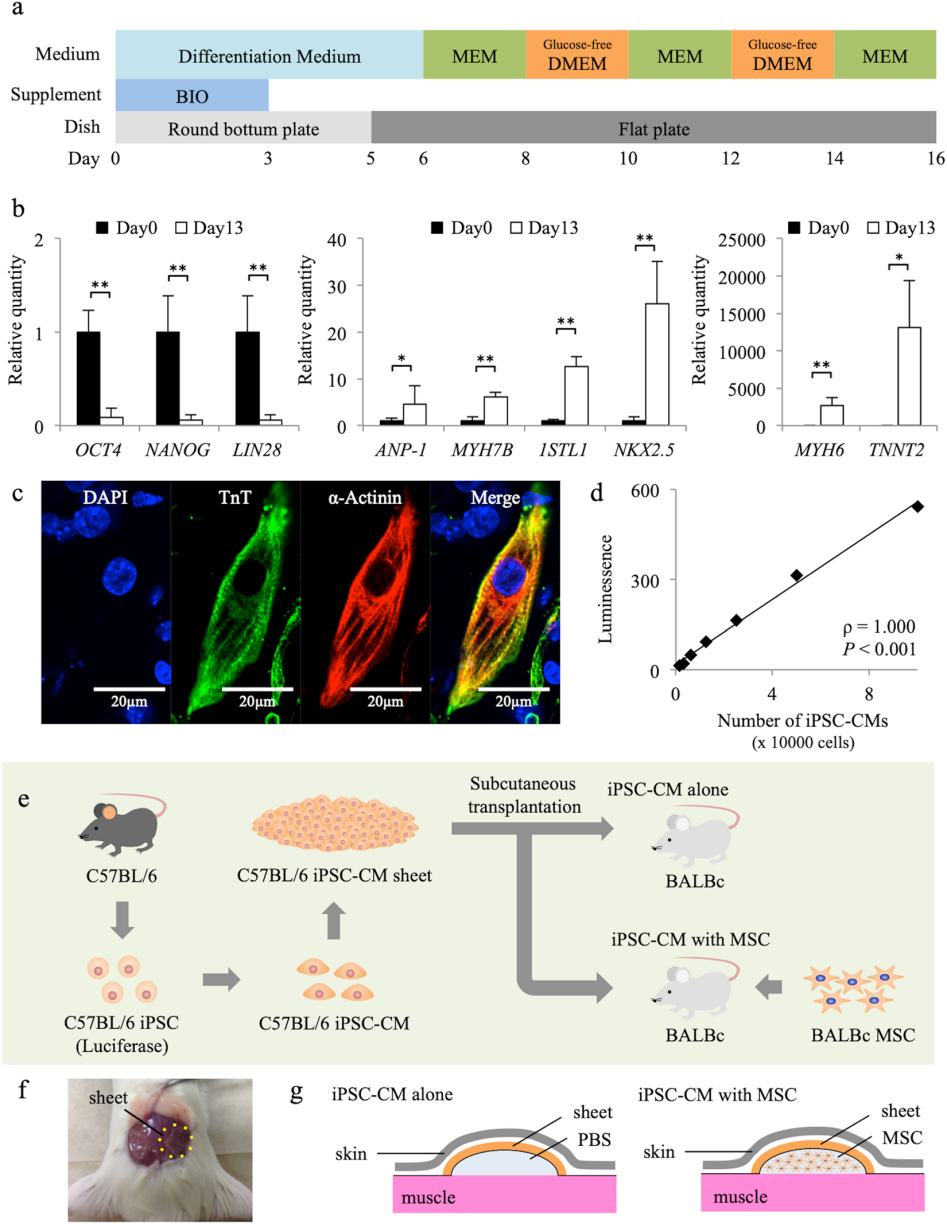
Experimental protocol and cardiomyogenic differentiation of murine induced pluripotent stem cells (iPSCs). (**a**) Cardiomyogenic differentiation protocol. (**b**) Expression of *OCT4*, *NANOG*, *LIN28*, *ANP-1*, *MYH7B*, *1STL1*, *NKX2.5*, *MYH6*, and *TNNT2* transcripts in iPSCs on days 0 and 13, as analysed by reverse transcription PCR. Results are relative to those at day 0 and are expressed as the means ± SE (n = 6 independent experiments). *P < 0.05, **P < 0.01. (**c**) Immunohistochemistry for troponin T (Alexa Fluor 488), alpha-actinin (Alexa Fluor 555), and DAPI in iPSC-derived cardiomyocytes (iPSC-CMs). Scale bar, 20 µm. (**d**) Positive association between the number of iPSC-CMs and luminescence (ρ = 1.000, *P* < 0.001). (**e**) Transplantation schema of murine iPSC-CM sheets derived from C57BL/6 mice into BALB/c mice. (**f**) Picture of subcutaneous transplantation of iPSC-CM sheets (C57BL/6) into the subcutaneous space of recipient mice (BALB/c). (**g**) Schema of subcutaneous transplantation of iPSC-CM sheets with and without mesenchymal stem cells (MSCs).

Undifferentiated murine iPSCs expressed *OCT4*, *NANOG*, and *LIN28*, and using a previously reported protocol, they were differentiated into cardiomyocytes that expressed cardiomyocyte marker genes such as *ANP-1*, *MYH7B*, *1STL1*, *NKX2.5*, *MYH6*, and *TNNT2*, with decreased *OCT4*, *NANOG*, and *LIN28* expression (Fig. 1a,b)^22–24^. Nearly all embryoid bodies exhibited self-beating at day 16 and the cells were positive for troponin T and alpha-actinin, indicating that they were iPSC-CMs (Fig. 1c, Movie SI). *In vitro* bioluminescence imaging (BLI) analysis of iPSC-CMs showed a linear relationship between cell number and signal (ρ = 1.000, *P* < 0.001), validating the use of this technique for the quantification of luciferase-expressing iPSC-CMs (Fig. 1d).

### Co-transplantation of syngeneic MSCs enhances the survival of transplanted allogeneic iPSC-CMs

**Figure 2:**
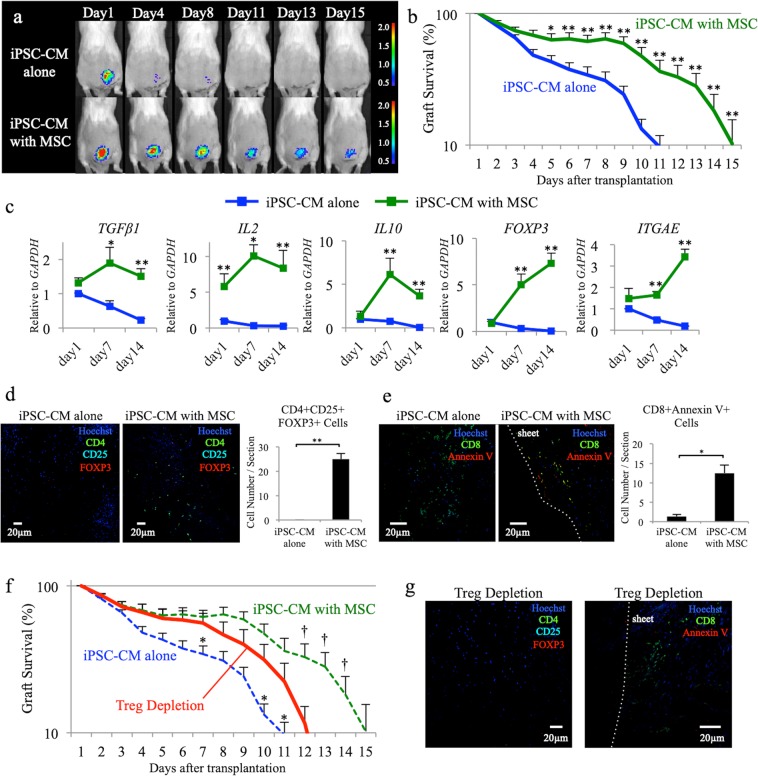
Allogeneic induced pluripotent stem cell-derived cardiomyocyte (iPSC-CM) sheet transplantation with or without syngeneic mesenchymal stem cells (MSCs). (**a**) Representative images of allogeneic iPSC-CM sheets, which were transplanted with or without syngeneic MSCs, using an *in vivo* imaging system. (**b**) Survival rate of allogeneic iPSC-CM sheets, which were transplanted with or without syngeneic MSCs, using an *in vivo* imaging system (n = 15, each); *P < 0.05, **P < 0.01. (**c**) Expression of *TGFβ1*, *IL2*, *IL10*, *FOXP3*, and *ITGAE* transcripts at the iPSC-CM sheet transplant site on day 1, 7, and 14, as analysed by reverse transcription polymerase chain reaction (n = 10, each); *P < 0.05, **P < 0.01. (**d**) Immunohistochemistry for CD4 (Alexa Fluor 488), CD25 (Alexa Fluor 555), FOXP3 (Alexa Fluor 647), and Hoechst33258 at the site of iPSC-CM sheet transplantation on day 7. Scale bars, 20 µm. The graph shows the number of CD4 + CD25 + FOXP3 + cells per section in each group. **P < 0.01. (**e**) Immunohistochemistry for CD8 (Alexa Fluor 488), Annexin V (Alexa Fluor 555), and Hoechst33258 at iPSC-CM sheet transplant site at day 7. Scale bars, 20 µm. The graph shows the number of CD8 + AnnexinV + cells per section in each group. *P < 0.05. (**f**) Survival rate of transplanted iPSC-CM sheets in regulatory T cell (Treg) depletion model using an *in vivo* imaging system (n = 15). (**g**) Immunohistochemistry for CD4 (Alexa Fluor 488), CD25 (Alexa Fluor 555), FOXP3 (Alexa Fluor 647), and Hoechst33258 at the site of iPSC-CM sheet transplantation on day 7 (left panel). Scale bars, 20 µm. Immunohistochemistry for CD8 (Alexa Fluor 488), Annexin V (Alexa Fluor 555), and Hoechst33258 at iPSC-CM sheet transplant site on day 7 (right panel). Scale bars, 20 µm.

We first subcutaneously transplanted allogeneic iPSC-CM sheets alone (iPSC-CM alone) or allogeneic iPSC-CMs sheets with syngeneic MSCs (iPSC-CM with MSC) (Fig. 1e–g). The survival of transplanted luciferase-expressing iPSC-CMs was monitored by serial BLI over time (Fig. 2a). The transplanted iPSC-CM sheets in mice treated with tacrolimus (Prograf; Astellas Pharma) survived for more than 2 weeks (Fig. SI). In the iPSC-CM alone group, cell survival was significantly decreased compared to that in the iPSC-CM with MSC group after day 4 (day 7: iPSC-CM alone, 34 ± 5%, iPSC-CM with MSC, 61 ± 7%, *P* = 0.008; day 10: iPSC-CM alone, 23 ± 5%, iPSC-CM with MSC, 47 ± 8%, *P* = 0.010; day 12: iPSC-CM alone, 7 ± 2%, iPSC-CM with MSC, 33 ± 8%, *P* = 0.004; day 14: iPSC-CM alone, 0 ± 0%, iPSC-CM with MSC, 18 ± 6%, *P* = 0.020), indicating that cell rejection occurred in the iPSC-CM alone group, whereas co-transplantation with MSCs suppressed allogeneic immune rejection (Fig. 2b).

Next, the T cell receptor repertoire was analysed using the spleens of mice administered iPSC-CMs alone or iPSC-CMs with MSCs on day 4 and 7 after sheet implantation, as well as those of a normal BALB/c mouse treated with a sham operation as a control. At day 4, in control, iPSC-CM alone, and iPSC-CM with MSC groups, no specific proliferated T cells were observed; however, these were identified in both iPSC-CM alone and iPSC-CM with MSC groups at day 7 (Fig. SII). These findings indicated that cell rejection might be one reason for the disappearance of the transplanted cells in both groups and that acquired immune rejection might function systemically after day 7 even when syngeneic MSCs are co-transplanted, but is suppressed locally in the presence of these cells.

### Co-transplantation with syngeneic MSCs suppresses the allogeneic immune reaction through Treg induction

The expression of *TGFβ1*, *IL2*, *IL10*, *FOXP3*, and *ITGAE* transcripts in the iPSC-CM with MSC group was higher than that in the iPSC-CM alone group at the site of iPSC-CM sheet transplantation for 2 weeks after sheet transplantation (Fig. 2c). Immunohistochemistry revealed that the number of CD4 + CD25 + FOXP3 + cells in the iPSC-CM with MSC group (25 ± 2 cells/section) was significantly higher than that in the iPSC-CM alone group (0 ± 0 cells/section; *P* = 0.006) at the site of transplantation on day 7 (Fig. 2d). Immunohistochemistry also revealed that the number of CD8 + AnnexinV + cells in mice administered iPSC-CMs with MSCs (13 ± 2 cells/section) was significantly higher than that in animals treated with iPSC-CMs alone (1 ± 1 cells/section; *P* = 0.020) at the site of transplantation on day 7 (Fig. 2e). Thus, co-transplanted MSCs induced CD4 + CD25 + FOXP3 + Tregs by secreting IL-2 and TGF-beta, as well as CD8 + T cell apoptosis, in the transplanted area, both of which might suppress immune reaction against the allogeneic iPSC-CM sheets.

To assess the immunosuppressive effect of MSCs via the induction of Tregs, the Treg depletion model, in which an anti-CD25 antibody is administered to mice before the co-transplantation of iPSC-CMs and MSCs, was used. The survival of transplanted iPSC-CMs in the Treg depletion model increased compared to that with iPSC-CMs alone (day 7: iPSC-CM alone, 34 ± 5%, Treg depletion, 56 ± 9%, *P* = 0.049; day 10: iPSC-CM alone, 10 ± 2%, Treg depletion, 32 ± 8%, *P* = 0.025) and decreased compared to that in the iPSC-CM with MSC group (day 12: iPSC-CM with MSC, 33 ± 8%, Treg depletion, 12 ± 4%, *P* = 0.020; day 14: iPSC-CM with MSC, 18 ± 6%, Treg depletion, 1 ± 1%, *P* = 0.014; Fig. 2f). Based on immunohistochemistry, CD4 + CD25 + FOXP3 + cells or CD8 + AnnexinV + cells were not detected at the transplant site in the Treg depletion model on day 7 after transplantation (Fig. 2g). These results indicated that the enhanced Treg population in the iPSC-CM with MSC group partially contributes to the extended survival of iPSC-CMs. Thus, mechanisms other than Tregs are required for the immunosuppressive effect of MSCs.

### Syngeneic MSCs affect activated lymphocytes through direct cell–cell contact

**Figure 3:**
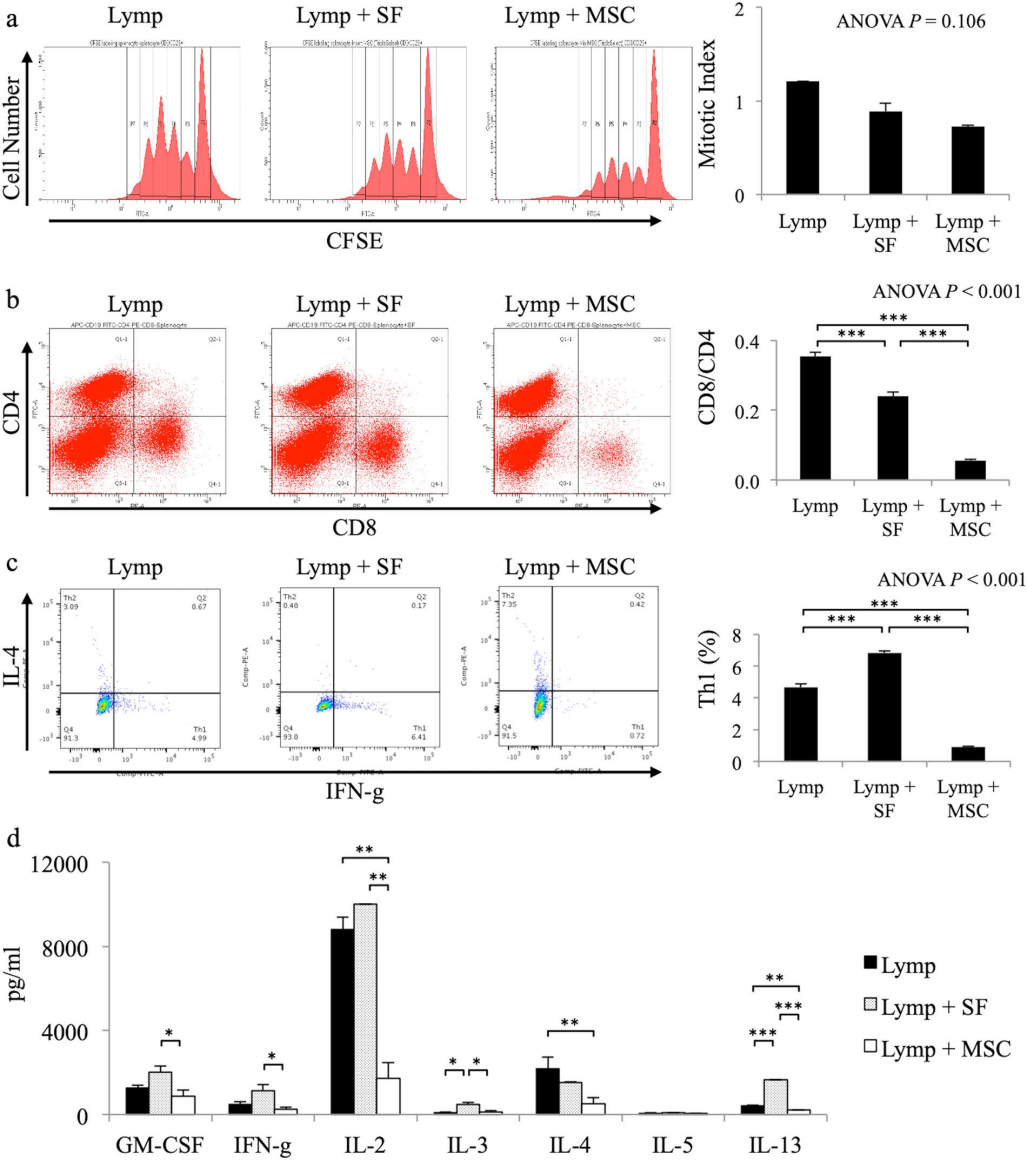
Direct effect of mesenchymal stem cells (MSCs) on lymphocytes. (**a**) Representative mixed lymphocyte reaction data for lymphocyte (lymp), lymphocyte + soluble factors from MSCs (lymp + SF), and lymphocyte + MSC (lymp + MSC) groups. The graph shows the mitotic index in each group (n = 3, each). (**b**) Representative flow cytometry data for lymp, lymp + SF, and lymp + MSC groups stained with anti-CD4 and anti-CD8 antibodies. The graph shows the ratio of CD8 + cells to CD4 + cells in each group (n = 3, each); ***P < 0.001. (**c**) Representative flow cytometry data for lymp, lymp + SF, and lymp + MSC groups stained with anti-CD4, anti-IL-4, and anti-IFN-g antibodies. The graph shows the percentage of IL4-IFG-g + cells among CD4 + cells in each group (n = 3, each); ***P < 0.001. (**d**) The concentration of cytokines in lymp, lymp + SF, and lymp + MSC groups (n = 3, each); *P < 0.05, **P < 0.01, ***P < 0.001.

To determine how MSCs exert their Treg-independent effect on iPSC-CM survival, we investigated the impact of syngeneic MSCs on activated lymphocytes using lymphocyte (lymp), lymphocytes + soluble factors from MSCs (lymp + SF), and lymphocytes + MSC (lymp + MSC) groups. First, the mitotic index, as a marker of the proliferation of activated lymphocytes, was analysed. There were no significant differences in mitotic indexes among the three groups (lymp: 1.2 ± 0.0, lymp + SF: 0.9 ± 0.1, lymp + MSC: 0.7 ± 0.0, ANOVA: *P* = 0.106; Fig. 3a). Second, the ratio of CD8/CD4 lymphocytes as an effective T cell marker was analysed. The ratio in the lymp + SF group (0.24 ± 0.01) was lower than that in the lymp group (0.35 ± 0.00, *P* < 0.001), but was higher than that in the lymp + MSC group (0.06 ± 0.00, *P* < 0.001; ANOVA: *P* < 0.001; Fig. 3b). These results suggested that effective T cells are suppressed by MSCs but not by soluble factors. Third, the proportion of IFN-g-positive cells among CD4-positive cells, as a type 1 helper T (Th1) cell marker, was analysed. The proportion of IFN-g-positive cells in the lymp group (4.7 ± 0.2%) was lower than that in the lymp + SF group (6.8 ± 0.1%, *P* < 0.001), but was significantly higher than that in the lymp + MSC group (0.9 ± 0.1%, *P* < 0.001; ANOVA: *P* < 0.001; Fig. 3c). These results suggested that the proportion of Th1 cells among CD4-positive cells were reduced by MSCs but not by soluble factors. Fourth, cytokine secretion in the culture medium was measured by enzyme-linked immunosorbent assay. The lymp + MSC group showed lower concentrations of GM-CSF (873 ± 291 pg/ml), IFN-g (248 ± 291 pg/ml), IL-2 (1708 ± 773 pg/ml), IL-3 (115 ± 82 pg/ml), and IL-13 (206 ± 3 pg/ml), compared to those in the lymp + SF group (GM-CSF: 2020 ± 277 pg/ml, *P* = 0.035; IFN-g: 1141 ± 286 pg/ml, *P* = 0.039; IL-2: 10021 ± 6 pg/ml, *P* = 0.001; IL-3: 489 ± 76 pg/ml, *P* = 0.023; IL-13: 1652 ± 13 pg/ml, *P* < 0.001). Furthermore, the lymp + MSC group exhibited lower concentrations of IL-2, IL-4 (523 ± 272 pg/ml), and IL-13 compared to those in the lymp group (IL-2: 8826 ± 564 pg/ml, *P* = 0.002; IL-4: 2189 ± 557 pg/ml, *P* = 0.038; IL-13: 413 ± 18 pg/ml, *P* = 0.001; Fig. 3d). These results suggested that cytokine secretion was suppressed by MSCs but not by soluble factors. Thus, syngeneic MSCs reduced the CD8/CD4 ratio, the proportion of Th1 cells among CD4-positive cells, and the secretion of several cytokines, resulting in the suppression of effective T cell functions.

## Discussion

**Figure 4:**
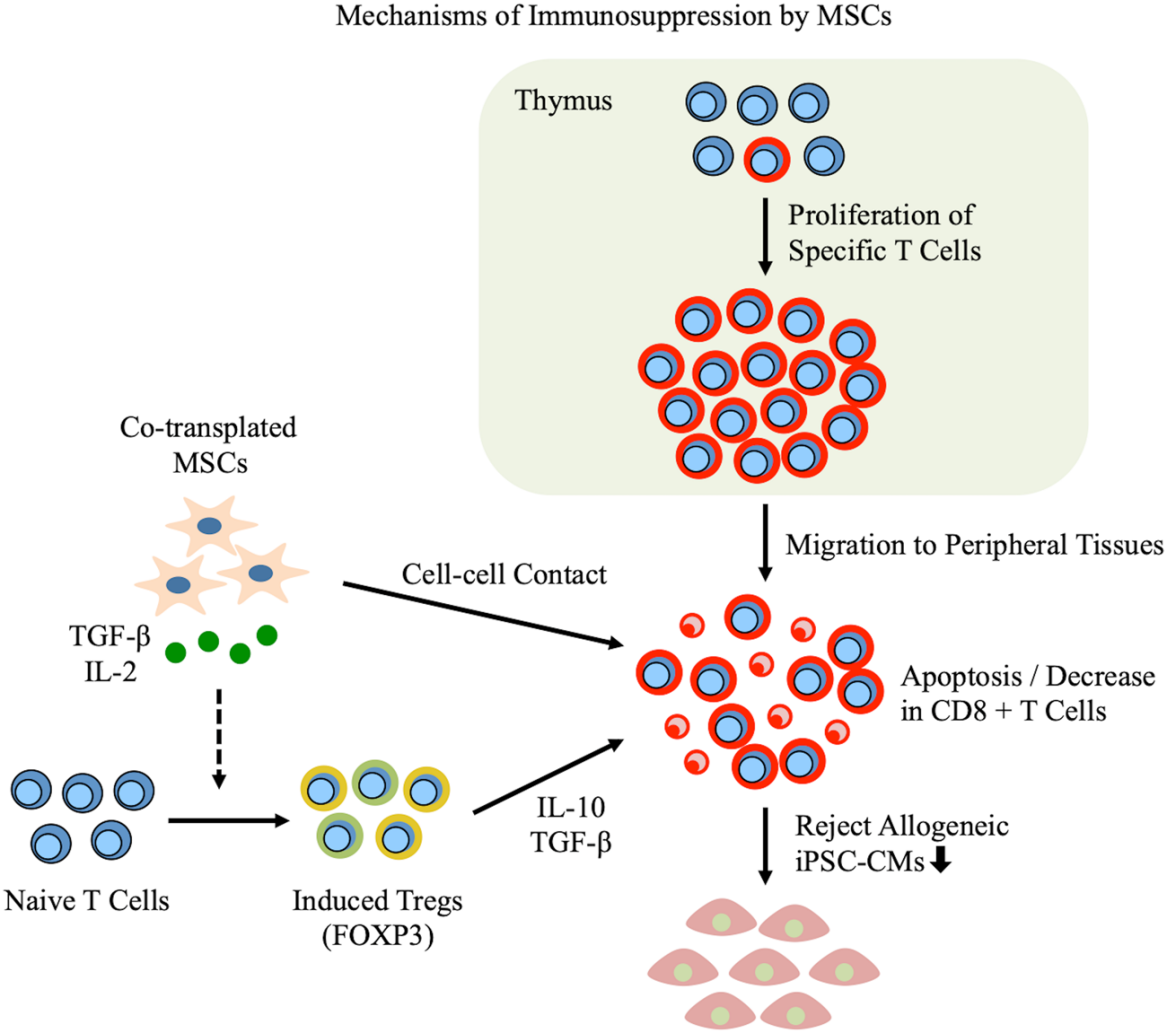
Schema of mechanisms of immunosuppression by mesenchymal stem cells (MSCs). The specific T cells proliferate in the thymus and migrate to peripheral tissues after the transplantation of allogeneic induced pluripotent stem cell-derived cardiomyocytes (iPSC-CMs), leading to acquired immune rejection. However, syngeneic MSC co-transplantation controls the immune rejection of allogeneic iPSC-CMs by mediating immune tolerance via regulatory T cells (Tregs), which was found to be induced by MSC-secreted IL-2 and TGF-beta, as well as cell–cell contact with activated T cells.

The major finding of this study was that the proliferation of specific T cells and adaptive immune rejection after allogeneic iPSC-CM transplantation occur despite the presence of syngeneic MSCs (Fig. 4). However, co-transplanted syngeneic MSCs were found to induce CD4 + CD25 + FOXP3 + Tregs by secreting IL-2 and TGF-beta, as well as CD8 + T cell apoptosis, in peripheral tissues. In addition, syngeneic MSCs reduced the CD8/CD4 ratio, the proportion of Th1 cells among CD4-positive cells, and the secretion of several cytokines through cell–cell contact. Accordingly, the subcutaneous engraftment of iPSC-CM sheets was enhanced by these mechanisms.

More than 90% of iPSCs differentiated into cardiomyocytes using this differentiation induction protocol, described in previous experiments^23,25^. The immunogenicity of undifferentiated iPSCs has been discussed, and several reports indicate that immunogenicity is reduced in the cells derived from iPSCs^26–29^. However, the reduction of immunogenicity varies depending on the cell type^27,30^. It is unclear whether co-transplanted MSCs suppress the immunogenicity of iPSCs or iPS-CMs, but challenges with respect to immunoreactions remain even if allogeneic immune rejection could be resolved.

It was reported that MSCs have well-defined immunomodulatory properties including the suppression of activated immune cells such as T cells, B cells, dendritic cells, natural killer cells, and macrophages^31–33^. Many studies have suggested that MSCs and immune cells have established two-way regulatory mechanisms; thus, the activation of MSC immunomodulatory properties requires the presence of derived proinflammatory cytokines from immune cells. Similarly, as a result of this activation, factors secreted by MSCs also regulate the immune response^33–35^. Syngeneic MSCs exert this immunosuppressive effect via the intravenous administration allogeneic cells, although co-transplantation with syngeneic MSCs into the peripheral tissue was reported to suppress allogeneic immune rejection, supporting the results of this study^36^. Co-transplanted MSCs only act locally at the transplant site avoiding non-specificity^36^. In addition, MSCs were reported to produce several pro-survival trophic factors that might play an important role in enhanced neighbour cell survival^37,38^. It was also suggested that MSCs, which were co-transplanted with iPSC-CMs, might have a positive effect on cardiomyocyte maturation and enhance their therapeutic effects for ischemic heart disease^39^. In addition, Treg cells not only have an immunosuppressive effect but also the ability to induce the proliferation of cardiomyocytes and endothelial cells, which might facilitate myocardial regenerative therapy^40–42^. Interestingly, previous studies have reported that MSCs express and secrete PD-1 and that Treg cells proliferate through PD1 signalling, suggesting an increase in the number of Treg cells after induction by MSCs^43,44^.

Interestingly, the present study demonstrated that the fraction of activated lymphocytes was affected by cell–cell contact between MSCs and lymphocytes. It was reported that MSCs inhibit the proliferation of CD4-positive and CD8-positive T cells^45^. Engela *et al*. demonstrated that MSCs can control CD8-positive T cells by inhibiting their proliferation, supporting the results of this study^46^. Unfortunately, we could not clarify the mechanism underlying the reduction in CD8-positive T cell and Th1 cell fractions, although previous reports have suggested the involvement of the immune checkpoint, wherein this ligand, appearing on the surface of MSCs, binds the inhibitory receptor of T cells to suppress T cell immunoreactions^47,48^. Among immune checkpoint molecules in T cells, only TIM3 is expressed at higher levels on CD8-positive T cells than on CD4-positive T cells, suggesting that it might play an important role in the immunosuppressive effect of cell–cell contact between MSCs and lymphocytes in this study^49^. In addition, Galactin-9, which is the ligand of TIM3, is expressed on the surface of MSCs and induces Th1 cell apoptosis by binding TIM3 on Th1 cells, leading to a reduction in CD8-positive cells^50,51^. Therefore, the immune checkpoint molecule TIM3 might be a major mechanism underlying the decrease in CD8-positive cells via cell–cell contact.

Furthermore, there is an increasing body of evidence indicating that tunnelling nanotubes facilitate molecular and subcellular structure exchange between neighbouring cells via the transfer of molecules and organelles such as calcium ions, prions, viral and bacterial pathogens, small lysosomes, and mitochondria^52–55^. Notably, Matulaa *et al*. reported the bidirectional exchange of cytoplasmic components between MSCs and T cells, which was found to be mediated by tunnelling nanotubes^56^. These findings support the contention that MSCs can affect the cell microenvironment through the transfer of cellular components to neighbouring T cells, in a manner that significantly contributes to cell regulation. Tunnelling nanotubes might thus contribute to the transfer of apoptosis signals to neighbouring CD8-positive cells and Th1 cells. Thus, MSCs might have multiple functions regarding the immunosuppression of neighbour cells, and our results could shed light on the mechanism underlying the immunosuppressive effect of MSCs.

In this study, the survival of transplanted iPSC-CMs was prolonged; however, the number of engrafted cells was less than 10% on the 15th day in the iPSC-CM with MSC group. One limitation of this study was that we did not label MSCs, but the reason as to why engraftment was not permanent might be partially attributed to a decrease in transplanted allogeneic MSCs due to immune rejection after the loss of their immunoprivilege. There are many merits associated with the use of allogeneic MSCs, and not syngeneic MSCs, considering clinical applications; allogeneic MSCs can be prepared well in advance, and are independent of the recipient’s condition including disease status and age^57^. However, allogeneic MSCs were found to be immunoprivileged early after implantation but gradually lost this phenotype^57,58^. Recently, methods to prolong the engraftment of allogeneic MSCs have also been reported and the further development of such methods might allow the long-term cell engraftment of allogeneic MSCs^59,60^.

Therapeutic treatment using allogeneic cells might be accompanied by the problem of immune rejection^10^. To avoid this, extracellular vesicles or cytokines secreted by the cells could be collected and used as a treatment^61–63^. However, this study aimed to establish a treatment that can provide not only the paracrine effect from the iPSC-CMs but can also increase the number of cardiomyocytes to enhance cardiac contraction. If the problem of immune rejection is resolved, the transplanted cells could continue to live in the recipient, secreting extracellular vesicles and cytokines and forming contracts with native cardiomyocytes, resulting in more effective treatment.

In conclusion, the co-transplantation of MSCs might control immune rejection against allogeneic iPSC-CMs after *in vivo* transplantation through the Treg induction and direct cell–cell contact; thus, this comprises a promising strategy for cardiomyogenesis therapy using allogeneic iPSCs for severe heart failure.

## Materials and Methods

Animal care procedures were consistent with the “Guide for the Care and Use of Laboratory Animals” (National Institutes of Health publication). Experimental protocols were approved by the Ethics Review Committee for Animal Experimentation of Osaka University Graduate School of Medicine (reference no. 25-025-045).

### *In vitro* cardiomyogenic differentiation of murine iPSCs

Luciferase-miPSCs (959A2-1-6) generated from C57BL/6 (B6) (CLEA) mouse embryonic fibroblasts were cultured in the absence of serum and feeder cells using ESGRO Complete PLUS Clonal Grade Medium (Millipore, Burlington, MA, USA). Cardiomyogenic differentiation of the iPSCs was performed as described, with modifications, followed by purification with glucose-free medium supplemented with lactic acid^22,23^; iPSCs (3 × 10^3^) were resuspended in 100-µl aliquots of differentiation medium [DM; Dulbecco’s Modified Eagle’s Medium (DMEM; Nacalai Tesque, Kyoto, Japan) containing 15% foetal bovine serum (FBS; Biofill, Melbourne, Victoria, Australia), 100 mmol/l non-essential amino acids (NEAA; Invitrogen, Carlsbad, CA, USA), 2 mmol/l L-glutamine (Invitrogen), and 0.1 mmol/l 2-mercaptoethanol (Invitrogen)] containing 0.2 mmol/l 6-bromoindirubin-3′-oxime (BIO; a glycogen synthase kinase-3β inhibitor to activate the Wnt-signalling pathway; Calbiochem, San Diego, CA, USA), and cultured in 96-well Corning Costar Ultra-Low attachment multiwell plates (MilliporeSigma, Burlington, MA) for 3 days. On day 3, an additional 100 µl of DM without BIO was added to each well. On day 5, individual embryoid bodies were transferred to 100-mm gelatine-coated dishes (250 per dish). On days 6, 7, 10, 11, 14, and 15, the medium was exchanged for serum-free Modified Eagle’s Medium (MEM; Invitrogen) with insulin transferrin selenium X (Invitrogen). On days 8, 9, 12, and 13, the medium was exchanged for glucose-free DMEM (no glucose, no pyruvate, Invitrogen) supplemented with 4 mmol/l lactic acid (FUJIFILM Wako Pure Chemical Corporation, Osaka, Japan) for the purification of cardiomyocytes. On day 16, the contracting cell clusters were dissociated, seeded on thermoresponsive dishes (5 × 10^6^ CMs/well; Upcell; CellSeed, Tokyo, Japan), and incubated at 37 °C for 2 days. At this time, they were transferred to 20 °C until the cells detached spontaneously to form scaffold-free cell sheets. The protocol and purification process are illustrated in Fig. 1a.

### Immunocytochemistry and analysis

Dissociated single cells or harvested tissues around the transplant site were fixed with 4% paraformaldehyde and labelled with primary antibodies, which was followed by incubation with fluorescence-conjugated secondary antibodies, counterstaining with 4′, 6-diamidino-2-phenylindole (DAPI; Vector Laboratories, Burlingame, CA, USA) or Hoechst33258 (Dojindo, Kumamoto, Japan), and finally analysis by confocal microscopy (FV1200, Olympus, Tokyo, Japan). The labelled cells were captured based on their fluorescence intensity. A list of the antibodies used can be found in Table SI.

### Quantitative reverse transcription PCR (RT-qPCR)

Total RNA from hiPSC-CMs cultured *in vitro* or from isolated tissue around the transplant site after cell sheet transplantation *in vivo* was isolated using the PureLink RNA Mini Kit (Thermo Fisher Scientific, Waltham, MA, USA) or RNeasy Fibrous Tissue Mini Kit (Qiagen, Hilden, Germany), respectively. RNA was reverse transcribed to cDNA using the SuperScript III reverse transcription kit (Thermo Fisher Scientific). RT-qPCR was performed using the Viia7 Real-Time PCR system (Thermo Fisher Scientific) in triplicate for each sample with TaqMan (Thermo Fisher Scientific) or SYBR green (Thermo Fisher Scientific) probes. The primers used for all PCR analyses can be found in Table SII. Expression levels were normalised to those of the housekeeping gene glyceraldehyde-3-phosphate dehydrogenase (*GAPDH*).

### Flow cytometry

Cells were dissociated with 0.25% trypsin-EDTA (Thermo Fisher Scientific), fixed with CytoFix fixation buffer (Becton Dickinson, Franklin Lakes, NJ, USA) for 20 min, permeabilised with Perm/Wash buffer (Becton Dickinson) at 20 °C for 10 min, and then incubated with primary antibody or isotype antibodies for 30 min. The labelled cells were washed with Perm/Wash buffer prior to incubation with the secondary antibody at room temperature for 30 min, and then assayed using a FACS Canto II (Becton Dickinson). A list of the antibodies used can be found in Table SI.

### T cell proliferation assay

After labelling with CFSE (Cayman Chemical Company, Ann Arbor, MI, USA), splenic lymphocytes (5 × 10^5^ cells/well) from BALB/c mice were incubated on 24-well plates coated with an anti-mouse CD3 antibody (clone; 145-2C11) with the same number of MSCs from BALB/c mice (MUBMX-01001, Cyagen Biosciences Inc, Santa Clara, CA, U.S.) (lymp + MSC) and without MSCs (lymp) using 1000 μl of RPMI 1640 containing 10% FBS. To assess the effects of MSC-secreted soluble factors, lymphocytes and MSCs were also co-cultured without direct cell–cell contact using transwell inserts (3.0-µm pore polycarbonate membrane; Corning Inc., Armonk, NY, USA) and the MSCs were removed before performing the assay (lymp + SF). An anti-mouse CD28 antibody (clone; 37.51, 100 ng/ml) was added to each well of the CD3-coated plate. After incubation for 72 h, the cells were dissociated with 0.25% trypsin-EDTA (Thermo Fisher Scientific) and stained with anti-mouse CD3 antibodies for 30 min, and then assayed using a FACS Canto II (Becton Dickinson). The mitotic index was provided as the sum of the mitotic events in all generations divided by the calculated number of original parent cells. A list of antibodies used can be found in Table SI.

### Bio-plex

The concentration of proteins secreted from cultured cells was measured using the Bio-Plex suspension array system (23-plex; Bio-Rad Laboratories) according to the manufacturer’s instructions. The culture media in the lymp, lymp + SF, and lymp + MSC groups were used for this experiment.

### Cell sheet transplantation

Adult male BALB/c mice (6–7 weeks old, 17–22 g) were generally anesthetised through the inhalation of isoflurane, and subjected to the subcutaneous transplantation of luciferase transgenic allogeneic iPSC-CM sheets with the intramuscular injection of 200 µl of PBS alone (CM) or 5 × 10^6^ MSCs in 200 µl of PBS (CM + MSC) at the site of iPSC-CM sheet transplant.

### Treg depletion model

BALB/c mice were administered intraperitoneal injections of 0.5 mg of an anti-CD25 antibody (clone PC61) 3 days before and 4, 11, and 18 days after the implantation of iPSC-CM sheets. PC61 administration promoted the long-lasting depletion/neutralisation of CD25 expression (longer than 2 weeks) in native mice, as previously demonstrated^64,65^. A list of antibodies used can be found in Table SI.

### Tacrolimus administration

An osmotic pump (ALZET Micro-Osmotic Pump model 1002; DURECT Corporation, Cupertino, CA, USA) filled with 1.5 mg/kg body weight of tacrolimus was subcutaneously implanted under the abdominal skin of the BALB/c mice to create an allogeneic model of immunosuppression. As one pump can continuously deliver the solution for 14 days, another new pump was added every 13 days to ensure the constant release of tacrolimus.

### *In vivo* imaging

Bioluminescent images were acquired using an *In Vivo* Imaging System (Perkin Elmer, Waltham, MA, USA). Animals were anesthetised with 2% isoflurane gas in oxygen, and 150 mg/kg D-luciferin (Summit Pharmaceuticals International Corporation, Tokyo, Japan) was injected intraperitoneally. Images were acquired 10 min after injection at the peak of the bioluminescence signal. Images were quantified by drawing a region of interest over the transplanted region, with data expressed as photon flux (p/s).

### Statistical analysis

Data are presented as the means with standard errors for continuous variables. Continuous variables were examined using the Student *t*-test. The one-way ANOVA test was used to compare values between more than two groups. When the one-way ANOVA test was significant, group differences were compared using the post hoc Tukey HSD test. Statistical analyses were performed using JMP®13 (SAS Institute Inc., Cary, NC, USA). Statistical significance was defined as *P* < 0.05.

## Supplementary information

SUPPLEMENTAL INFORMATION.

## Data Availability

The authors confirm that the data supporting the findings of this study are available within the article and its supplementary materials.
