# Publisher Correction: Syngeneic Mesenchymal Stem Cells Reduce Immune Rejection After Induced Pluripotent Stem Cell-Derived Allogeneic Cardiomyocyte Transplantation

**DOI:** 10.1038/s41598-020-67129-9

**Published:** 2020-06-17

**Authors:** Shohei Yoshida, Shigeru Miyagawa, Toshihiko Toyofuku, Satsuki Fukushima, Takuji Kawamura, Ai Kawamura, Noriyuki Kashiyama, Yuki Nakamura, Koichi Toda, Yoshiki Sawa

**Affiliations:** 1 Department of Cardiovascular Surgery, Osaka University Graduate School of Medicine, 2-2 Yamadaoka, Suita, Osaka 565-0871 Japan; 2 Department of Immunology and Molecular Medicine, Osaka University Graduate School of Medicine, Suita, Osaka Japan

Correction to: *Scientific Reports* 10.1038/s41598-020-58126-z, published online 12 March 2020

This article contains an error in the Discussion section.

“In this study, the survival of transplanted iPSC-CMs was prolonged; however, the number of engrafted cells was less than 10% on the 15th day in the iPSC-CM with MSC group. One limitation of this study was that we did not label MSCs, but the reason as to why engraftment was not permanent might be partially attributed to a decrease in transplanted allogeneic MSCs due to immune rejection after the loss of their immunoprivilege. There are many merits associated with the use of allogeneic MSCs, and not syngeneic MSCs, considering clinical applications; allogeneic MSCs can be prepared well in advance, and are independent of the recipient’s condition including disease status and age^57^. However, allogeneic MSCs were found to be immunoprivileged early after implantation but gradually lost this phenotype^57,58^. Recently, methods to prolong the engraftment of allogeneic MSCs have also been reported and the further development of such methods might allow the long-term cell engraftment of allogeneic MSCs^59,60^.”

should read:

“There are many merits associated with the use of allogeneic MSCs, and not syngeneic MSCs, considering clinical applications; allogeneic MSCs can be prepared well in advance, and are independent of the recipient’s condition including disease status and age^57^. However, allogeneic MSCs were found to be immunoprivileged early after implantation but gradually lost this phenotype^57,58^. Recently, methods to prolong the engraftment of allogeneic MSCs have also been reported and the further development of such methods might allow the long-term cell engraftment of allogeneic MSCs^59,60^.”

