# Supplementary material for: Syngeneic Mesenchymal Stem Cells Reduce Immune Rejection After Induced Pluripotent Stem Cell-Derived Allogeneic Cardiomyocyte Transplantation
Source: Sci Rep. 2020 Mar 12;10:4593. doi: 10.1038/s41598-020-58126-z (PMC7067786; doi:10.1038/s41598-020-58126-z)
Supplement: Supplementary file 1 — SUPPLEMENTAL INFORMATION. [file 41598_2020_58126_MOESM1_ESM.docx]

**Syngeneic Mesenchymal Stem Cells Reduce Immune Rejection After Induced Pluripotent Stem Cell-Derived Allogeneic Cardiomyocyte Transplantation**

Shohei Yoshida M.D., Ph.D., Shigeru Miyagawa M.D., Ph.D., Toshihiko Toyofuku M.D., Ph.D., Satsuki Fukushima M.D., Ph.D., Takuji Kawamura M.D., Ph.D., Ai Kawamura M.D., Ph.D., Noriyuki Kashiyama M.D., Ph.D., Yuki Nakamura M.D., Ph.D., Koichi Toda M.D., Ph.D., Yoshiki Sawa M.D., Ph.D.

SUPPLEMENTAL INFORMATION

Table SI. Lists of primary antibodies used in this study.

Table SII. Lists of primers used in this study.

Figure SI. Survival rate of transplanted induced pluripotent stem cell-derived cardiomyocytes (iPSC-CMs) with tacrolimus.

Figure SII. T cell receptor repertoire analysis.

Movie SI. Beating of murine induced pluripotent stem cell-derived cardiomyocyte (iPSC-CMs) on day 16.

**SUPPLEMENTAL TABLE**

**Table SI. List of primary antibodies used in this study.**

|  | Antibodies | Source |
| --- | --- | --- |
| **For immunocytochemistry and immunohistochemistry** | |  |
|  | ms CD4 | Abcam, ab25475 |
|  | ms CD8 | Abcam, ab22378 |
|  | ms CD25 | R&D, AF2438 |
|  | ms FOXP3 | Abcam, ab54501 |
|  | ms Annexin V | Abcam, ab14196 |
|  | TnT | Thermo scientific, MS-295-P1 |
|  | α-actinin | Sigma-Aldrich, A7732 |
| **For flow cytometry** | |  |
|  | ms CD4 | BD Bioscience, 563729 |
|  | ms CD8 | BD Bioscience, 553033 |
|  | IL-4 | BD Bioscience, 554435 |
|  | IFN-g | eBioscience |
| **For T cell proliferation assay** | |  |
|  | ms CD3 | BD Bioscience, 553057 |
|  | ms CD28 | Affymetrix, 16-0281-85 |
| **For Treg depletion model** | |  |
|  | ms CD25 | BioLegend, 102014 |

**Table SII. Lists of primers used in this study.**

| **Gene Name** | **Primers** |  |
| --- | --- | --- |
| *GAPDH* | taqman | Mm99999915_g1 |
| *FOXP3* | taqman | Mm00475162_m1 |
| *ITGAE* | taqman | Mm00434443_m1 |
| *TGFB1* | taqman | Mm01178820_m1 |
| *MYH7B* | taqman | Mm01249941_m1 |
| *IL2* | taqman | Mm00434256_m1 |
| *IL10* | taqman | Mm00439614_m1 |
| *TNNT2* | taqman | Mm01290256_m1 |
| *GAPDH* | SYBR | F: 3′-CCAGTATGACTCCACTCACG-5′ R: 5′-GACTCCACGACATACTCAGC-3′ |
| *LIN28* | SYBR | F: 3′-CTGCTGTAGCGTGATGGTTGA-5′ R: 5′-CCACCCAATGTGTTCTATTGCA-3′ |
| *NANOG* | SYBR | F: 3′-TCGCCATCACACTGACATGA-5′ R: 5′-TGTGCAGAGCATCTCAGTAGCA-3′ |
| *OCT4* | SYBR | F: 3′-TTTAACCCCAAAGCTCCAGG-5′ R: 5′-GGCTCTCCCATGCATTCAA-3′ |
| *ANP-1* | SYBR | F: 3′-AAAGAAACCAGAGTGGGCAGAG-5′ R: 5′-CCAGGGTGATGGAGAAGGAG-3′ |
| *NKX2.5* | SYBR | F: 3′-CAAGTGCTCTCCTGCTTTCC-5′ R: 5′-GGCTTTGTCCAGCTCCACT-3′ |
| *ISTL1* | SYBR | F: 3′-TTTCCCTGTGTGTTGGTTGC -5′ R: 5′-TGATTACACTCCGCACATTTCA-3′ |
| *MYH6* | SYBR | F: 3′-GAGATTTCTCCAACCCAG-5′ R: 5′-CCAGGGTGATGGAGAAGGAG-3′ |

**SUPPLEMENTAL FIGURE**

**Figure SI. Survival rate of transplanted induced pluripotent stem cell-derived cardiomyocytes (iPSC-CMs) with tacrolimus.**


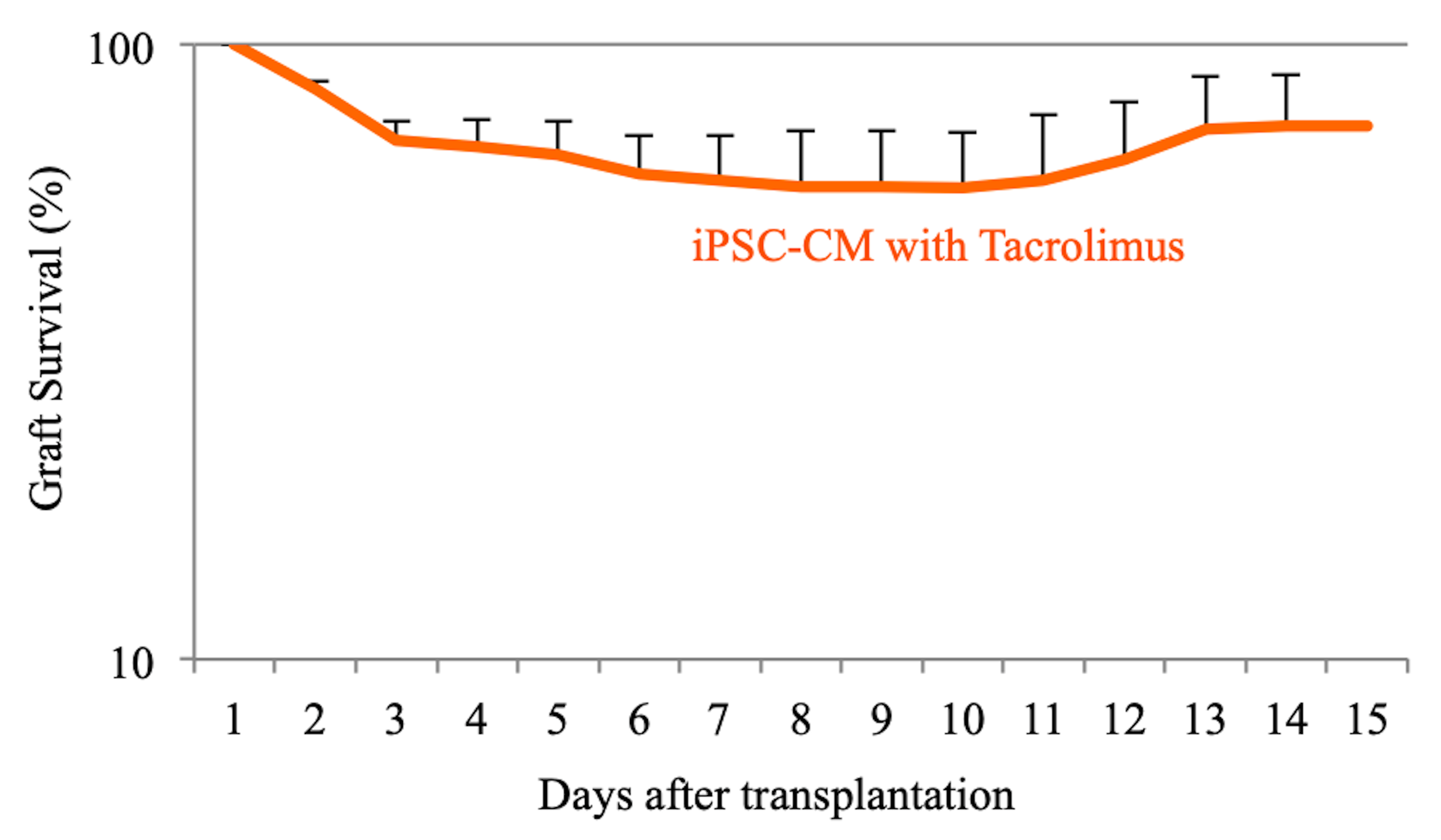
Survival rate of allogeneic iPSC-CM sheets, which were transplanted with tacrolimus, using an *in vivo* imaging system (n = 15).

**Figure SII. T cell receptor repertoire analysis.**


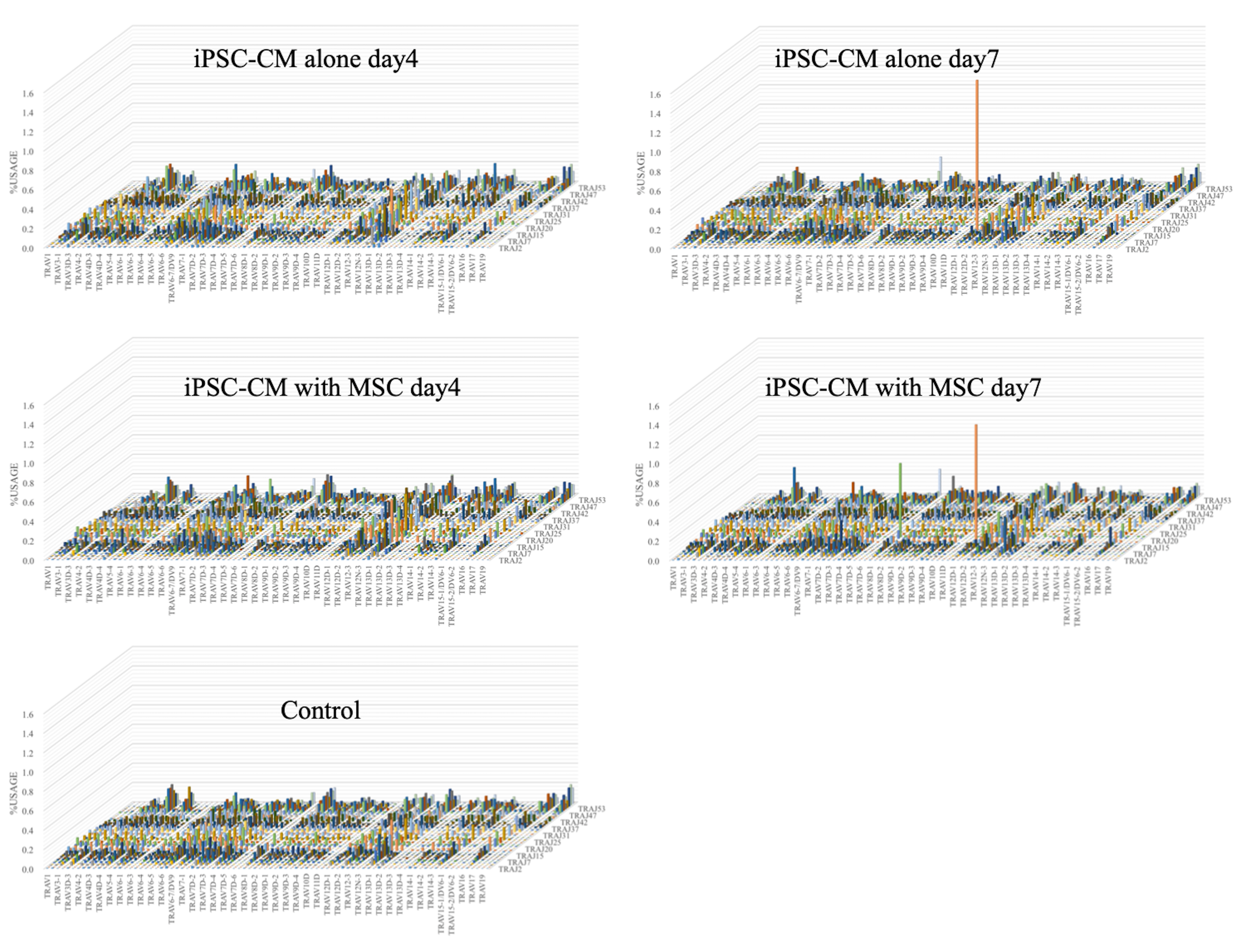
The T cell receptor repertoire was analyzed using the spleens of induced pluripotent stem cell-derived cardiomyocyte (iPSC-CM) alone and iPSC-CM with mesenchymal stem cell (MSC) groups on day 4 and 7 after iPSC-CM sheet implantation and in a normal BALB/c mouse subjected to a sham operation as a control. Control and iPSC-CM alone and iPSC-CM with MSC groups on day 4 did not exhibit specific proliferated T cells, whereas specific T cells were identified in both iPSC-CM alone and iPSC-CM with MSC groups on day 7.

**SUPPLEMENTAL MOVIE**

**Movie SI. Beating of murine induced pluripotent stem cell-derived cardiomyocyte (iPSC-CMs) on day 16.**
